# Antimicrobial sales profile in Brazil from 2014 to 2021: analysis of records from the National System of Controlled Products Management

**DOI:** 10.1590/1980-549720250040

**Published:** 2025-08-04

**Authors:** Jakeline Ribeiro Barbosa, Giovanny Vinícius Araújo de França, Aurélio Matos Andrade, Beatriz Torres Araújo, Cláudio Maierovitch Pessanha Henriques, Mariana Pastorello Verotti

**Affiliations:** I Fundação Oswaldo Cruz, Universidade Aberta do Sistema Único de Saúde, Executiva Secretariat - Brasília (DF), Brazil.; II Ministry of Health, Secretariat of Science, Technology and Innovation and the Health Economic-Industrial Complex, Department of Science and Technology - Brasília (DF), Brazil.; III Fundação Oswaldo Cruz, Evidence for Health Policy and Technology Program - Brasília (DF), Brazil.; IV Fundação Oswaldo Cruz, Epidemiology and Health Surveillance Center - Brasília (DF), Brazil.

**Keywords:** Anti-infective agents, Products commerce, Information systems, Brazil, Drug resistance, microbial

## Abstract

**Objective:**

To analyze the antimicrobial dispensing profile in Brazil from 2014 to 2021 based on records from the Brazilian National System of Controlled Products Management (SNGPC) of the Brazilian Health Regulatory Agency (Anvisa).

**Methods:**

A descriptive, time-series ecological study was carried out using data from private pharmacies and drugstores on the sale of antimicrobial drugs. Drugs sold from January 2014 to November 2021 were included, with analysis of the variables of month, year, municipality, state, active ingredient, prescriber’s professional council, and patient’s sex and age.

**Results:**

During the study period, 532,518,866 sales of special control drugs were recorded in SNGPC, 66.8% of which were antimicrobials. There was an increase in sales up to 2019, with a decrease in 2020, during the COVID-19 pandemic, and a new increase in 2021. The Southeast and Northeast regions concentrated the highest sales, with the Southeast leading. Sales were higher among women, especially in the 30 to 44.9 age group, with an increase in sales among patients aged 60 or older. The best-selling antimicrobials were amoxicillin, azithromycin, ciprofloxacin and cephalexin.

**Conclusion:**

The study reveals differences in consumption by region, sex, and age, and it highlights the importance of educational interventions for the responsible use of antimicrobials. It also points out that the suspension of mandatory data registration in SNGPC may compromise the monitoring necessary to combat bacterial resistance and improve public health in Brazil.

## INTRODUCTION

The use of antimicrobials is a milestone in the history of medicine, providing effective treatment for a wide range of infections, especially bacterial infections, which were previously the most common causes of morbidity and mortality^1^ ^,^ ^2^ ^,^ ^3^. However, the alarming growth of antimicrobial resistance (AMR) currently represents one of the greatest threats to global health. AMR not only limits available therapeutic options, but also increases health care costs and increases the burden of disease worldwide^4^ ^,^ ^5^. The situation regarding antimicrobial consumption varies significantly around the world, reflecting differences in prescribing practices, access to medicines and health policies^6^ ^,^ ^7^. In developing countries, such as Brazil, the issue is particularly complex because of socioeconomic and structural inequality, in addition to specific challenges related to the implementation of policies for the rational use of antimicrobials^8^ ^,^ ^9^.

In the Brazilian context, AMR has become a growing concern^10^ ^,^ ^11^ ^,^ ^12^. Epidemiological studies have shown worrisom levels of bacteria resistant to commonly used antimicrobials, such as penicillins and cephalosporins, both in hospital settings and in the community, compromising the effectiveness of treatments, increasing the severity of infections and the duration of the disease, as well as increasing health care costs^9^ ^,^ ^12^ ^,^ ^13^. The spread of AMR in health care facilities, such as hospitals and clinics, as well as in communities, highlights the urgent need for coordinated strategies to control and mitigate this public health problem^11^ ^,^ ^14^.

Accordingly, as of 2011, the Brazilian Health Regulatory Agency (Anvisa) established the control of antimicrobial-based drugs, and in 2014, their registration became mandatory in the National System of Controlled Products Management (SNGPC), as it was already for drugs subject to special control. SNGPC was established to monitor and regulate the dispensing of medicines, including antimicrobials, in private pharmacies and drugstores throughout Brazil^15^ ^,^ ^16^.

Over the years, SNGPC has played a crucial role in regulating and monitoring the dispensing of antimicrobials in the country. Since its implementation, SNGPC has provided a powerful infrastructure for collecting and analyzing data on the distribution and use of these drugs in pharmacies and drugstores throughout the country. This system enables a detailed assessment of prescribing and dispensing patterns, as well as the production of essential information for the development of evidence-based health policies^17^ ^,^ ^18^ ^,^ ^19^.

The analysis and monitoring of antimicrobial prescription and consumption habits in the country has improved the understanding of trends in inappropriate use, given that the National Drug Policy (PNM) is still in its infancy regarding the dispensing of antimicrobials in Brazil. This also interferes with public health policies aimed at promoting the rational use of these drugs, which contribute to regulatory decisions and educational actions on prescription use. The aim of the present study was to analyze the antimicrobial dispensing profile in Brazil during the period from 2014 to 2021.

## METHODS

A descriptive, time-series ecological study was carried out on the basis of SNGPC databases, made publicly available by Anvisa through the Brazilian Open Data Portal (PBDA), at the electronic address: https://dados.gov.br/dados/organizacoes/visualizar/agencia-nacional-de-vigilancia-sanitaria-anvisa. The data for this study were obtained from SNGPC’s records of dispensing of industrialized medicines, referring to the class of antimicrobials, from private pharmacies and drugstores. The most recent update of the PBDA occurred on August 8, 2023, and the data are available under the Creative Commons Attribution license:

- File with system documentation and variable dictionary;
- Another 95 files in Comma-Separated Values (CSV) format, containing sales of manufactured medicines, month by month, from January 2014 to November 2021.

According to Collegiate Board Resolution (RDC) 586/2021, the deadlines for transmitting data on the sale of drugs subject to special control to the national SNGPC database were temporarily suspended, becoming optional as of October 5, 2021. As of December 23, 2022, access to the system was completely interrupted, with the justification that a technological solution would be implemented. Therefore, in the period considered for analysis, from January 1, 2024 to November 30, 2021, data for the months of September 2014 and July 2020 were not available.

The 95 files were individually extracted from the PBDA website, in “.csv” format and converted to “*.dta” format, to enable analysis using Stata^®^ version 12.0. In each file, records with the prescription type variable equal to “5 - Antimicrobial Prescription in 2 copies” were selected to maintain only antimicrobial sales. Subsequently, the files were grouped by year to optimize processing and analysis.

The following variables were considered: month and year of sale of the medication; city and federative unit of the address of the pharmacy or drugstore where the sale occurred; name of the active ingredient of the manufactured medication, as registered with Anvisa; professional association of the professional who prescribed the medication sold; federative unit of the professional association of the prescribing professional; patient’s sex (male or female); and patient’s age in months or years.

Regarding the missing data for the months of September 2014 and July 2020, the ipolate command of Stata^®^ was used, which considers the historical series and performs the imputation using linear interpolation to estimate the missing points, on the basis of the known points of the series. Considering that sending data to SNGPC became optional as of October 5, 2021, the same technique was used to impute values for the months of November 2021.

This study was carried out in accordance with Resolutions No. 466/2012 and 580/2018 of the National Health Council (CNS). This research is part of a larger project titled «Brazilian Scenario of Antimicrobial Use and the Resistance Profile Identified in SUS», which was approved by the Research Ethics Committee under CAAE 42456920.6.0000.8027 and funded by the National Council for Scientific and Technological Development (CNPq)/Regional Management of Brasília/Fiocruz (call for proposals 41/2018, Process No. 440206/2019-7).

### Data Availability Statement

The data are available in: https://dados.gov.br/dados/conjuntos-dados/venda-de-medicamentos-controlados-e-antimicrobianos---medicamentos-industrializados.

## RESULTS

**Figure 1.**
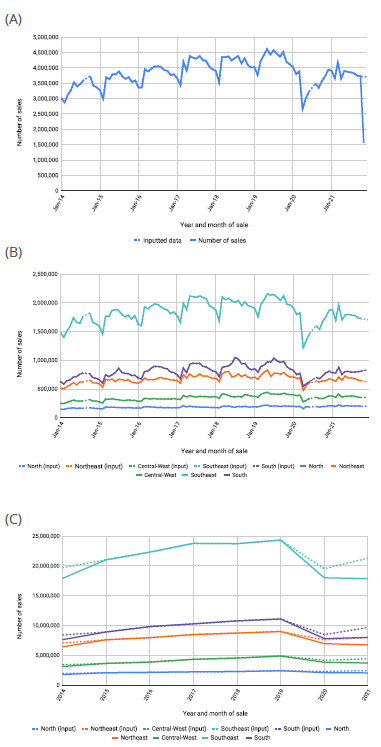
Number of sales of manufactured medicines containing an antimicrobial as an active ingredient, (A) by year/month of sale, (B) by year/month of sale and region and (C) by year of sale and region, comparing the series with observed values ​​with the series including inputted values, Brazil, 2014 to 2021*.

From January 2014 to November 2021, 532,518,866 sales of special control and antimicrobial manufactured drugs were recorded in SNGPC. Of these, 355,683,685 had a prescription type equal to “5 - Antimicrobial Prescription in 2 copies”, corresponding to 66.8% of the total. Figure 1 shows the historical series of antimicrobial sales by year and month of sale, total (A) and stratified by region (B), as well as the series with the consolidated sales by year and region (C). In the graphs, it is possible to compare the raw data obtained in relation to the series considering the imputations made.

*Sales data for the months of September 2014 and July 2020 are not available.

A linear trend of growth in sales was observed over the years from 2014 to 2019 and apparent seasonality, with a notable increase in the middle of the year in the South and Southeast regions, during the winter, and a reduction in all regions at the beginning of each year. The aforementioned growth until 2019 was interrupted, with a sharp drop in 2020, the year in which the COVID-19 pandemic began. In November 2021, the imputed data suggests that the data observed in this period was significantly underestimated, with a slight drop expected in the months of November and December, following the trend of decreasing sales observed from June 2021 (Figure 1A).

The Southeast and Northeast regions stand out, as they concentrated most of the sales in the analyzed period, with the Southeast region ranking first, being consistently higher than the other regions throughout the series (Figure 1B and 1C). The ordering of sales volumes by geographic region corresponds directly to the order of number of inhabitants. Considering the data aggregated by year and by region, different patterns are noted in the series with and without imputed values, especially in the Southeast and Northeast regions. On the basis of imputation analyses, there was a sharp drop in sales in 2020 and a trend of significant increase in sales in 2021, which was not possible to identify through the raw data.

The Southeast and South regions showed high proportion of municipalities with sales records in SNGPC throughout the period, with little variability. The states of Espírito Santo and Rio de Janeiro reached 100% of municipalities with records in 2021; Rio de Janeiro had 100% coverage between 2015 and 2021. The Central-West region also showed high coverage, but with greater variability, going from 87.0% in 2014 to 95.0% in 2021. The states of Goiás, Mato Grosso do Sul and Mato Grosso had homogeneous coverage among themselves throughout the series (Table 1).

**Table 1.** Total number and percentage of municipalities in each federative unit (FU) that registered sales of antimicrobials in the National System of Management of Controlled Products (SNGPC), stratified by year, Brazil, 2014 to 2021.

Region/FU	Total cities	2014		2015		2016		2017		2018		2019		2020		2021	
		n	%	n	%	N	%	n	%	n	%	n	%	n	%	n	%
North	450	200	44.4	198	44.0	215	47.8	225	50.0	232	51.6	251	55.8	252	56.0	275	61.1
Acre	22	18	81.8	18	81.8	17	77.3	18	81.8	17	77.3	17	77.3	17	77.3	18	81.8
Amapá	16	2	12.5	2	12.5	2	12.5	8	50.0	5	31.3	5	31.3	3	18.8	4	25.0
Amazonas	62	11	17.7	10	16.1	12	19.4	11	17.7	10	16.1	15	24.2	16	25.8	20	32.3
Pará	144	68	47.2	64	44.4	65	45.1	64	44.4	66	45.8	70	48.6	68	47.2	74	51.4
Rondônia	52	46	88.5	45	86.5	47	90.4	48	92.3	49	94.2	50	96.2	51	98.1	51	98.1
Roraima	15	5	33.3	6	40.0	8	53.3	9	60.0	10	66.7	10	66.7	11	73.3	13	86.7
Tocantins	139	50	36.0	53	38.1	64	46.0	67	48.2	75	54.0	84	60.4	86	61.9	95	68.3
Northeast	1,794	1,233	68.7	1,282	71.5	1,309	73.0	1,346	75.0	1,348	75.1	1,371	76.4	1,411	78.7	1,437	80.1
Alagoas	102	65	63.7	73	71.6	77	75.5	81	79.4	83	81.4	87	85.3	87	85.3	88	86.3
Bahia	417	301	72.2	324	77.7	342	82.0	348	83.5	359	86.1	370	88.7	375	89.9	375	89.9
Ceará	184	145	78.8	144	78.3	146	79.3	149	81.0	149	81.0	146	79.3	153	83.2	156	84.8
Maranhão	217	107	49.3	106	48.8	103	47.5	112	51.6	111	51.2	111	51.2	114	52.5	118	54.4
Paraíba	223	175	78.5	187	83.9	187	83.9	196	87.9	196	87.9	197	88.3	202	90.6	210	94.2
Pernambuco	185	158	85.4	155	83.8	155	83.8	159	85.9	159	85.9	162	87.6	162	87.6	161	87.0
Piauí	224	100	44.6	102	45.5	99	44.2	97	43.3	87	38.8	91	40.6	107	47.8	111	49.6
Rio Grande do Norte	167	131	78.4	138	82.6	145	86.8	148	88.6	149	89.2	149	89.2	148	88.6	152	91.0
Sergipe	75	51	68.0	53	70.7	55	73.3	56	74.7	55	73.3	58	77.3	63	84.0	66	88.0
Southeast	1,668	1,543	92.5	1,571	94.2	1,588	95.2	1,600	95.9	1,601	96.0	1,611	96.6	1,615	96.8	1,621	97.2
Espírito Santo	78	74	94.9	74	94.9	74	94.9	75	96.2	76	97.4	77	98.7	78	100.0	78	100.0
Minas Gerais	853	776	91.0	792	92.8	804	94.3	813	95.3	819	96.0	825	96.7	825	96.7	829	97.2
Rio de Janeiro	92	91	98.9	92	100.0	92	100.0	92	100.0	92	100.0	92	100.0	92	100.0	92	100.0
São Paulo	645	602	93.3	613	95.0	618	95.8	620	96.1	614	95.2	617	95.7	620	96.1	622	96.4
Central-West	501	436	87.0	447	89.2	451	90.0	454	90.6	462	92.2	470	93.8	471	94.0	476	95.0
Federal District*	35	21	60.0	22	62.9	22	62.9	22	62.9	22	62.9	23	65.7	26	74.3	26	74.3
Goiás	246	218	88.6	224	91.1	230	93.5	230	93.5	233	94.7	238	96.7	237	96.3	239	97.2
Mato Grosso	141	127	90.1	132	93.6	129	91.5	131	92.9	134	95.0	135	95.7	134	95.0	138	97.9
Mato Grosso do Sul	79	70	88.6	69	87.3	70	88.6	71	89.9	73	92.4	74	93.7	74	93.7	73	92.4
South	1,191	1,099	92.3	1,117	93.8	1,124	94.4	1,140	95.7	1,140	95.7	1,146	96.2	1,146	96.2	1,155	97.0
Paraná	399	373	93.5	375	94.0	378	94.7	386	96.7	384	96.2	382	95.7	382	95.7	388	97.2
Rio Grande do Sul	497	454	91.3	466	93.8	466	93.8	468	94.2	470	94.6	472	95.0	474	95.4	475	95.6
Santa Catarina	295	272	92.2	276	93.6	280	94.9	286	96.9	286	96.9	292	99.0	290	98.3	292	99.0

*The Federal District (FD) is divided into administrative regions and not cities.

The North region had the lowest coverage, ranging from 44.4% in 2014 to 61.1% in 2021. The states with the fewest antimicrobial sales records in SNGPC were Amapá and Amazonas, where 25 and 32.3% of municipalities had sales records in 2021, respectively. The state of Roraima showed a significant increase in the number of municipalities with records, rising from 5 in 2014 to 13 in 2021 out of a total of 15 municipalities (Table 1).

Sales were higher for females in the period 2014-2021, with percentages ranging from 55.1% to 55.7%. The distribution of sales by patient age was predominant in the age group of 30 to 44.9 years, varying between 24.9% in 2014 and 28.2% in 2021. The category «60 years or older» showed growth in consumption over the years, going from 16.0% in 2014 to 19.9% in 2021, which corresponds approximately to the proportion of people in this age group in the population. The age group of people under 15 years old was the one that showed the sharpest decrease in prescriptions between 2019 and 2020, with a slight increase in 2021 compared to the previous year (Table 2).

**Table 2.** Number of sales of manufactured medicines containing an antimicrobial as an active ingredient by year of sale, according to the prescriber’s council and sex and age of the patient, Brazil, 2014 to 2021^!^.

Variables	Year of sale							
	2014*	2015	2016	2017	2018	2019	2020*	2021
	n (%)	n (%)	n (%)	n (%)	n (%)	n (%)	n (%)	n (%)
Patient’s sex**								
Male	16,263,873 (44.9)	19,145,755 (45.0)	20,266,780 (44.8)	21,499,749 (44.6)	21,838,691 (44.4)	22,702,196 (44.7)	16,719,276 (44.3)	17,680,913 (45.4)
Female	19,967,350 (55.1)	23,419,952 (55.0)	25,021,209 (55.3)	26,744,166 (55.4)	27,313,786 (55.6)	28,060,636 (55.3)	21,044,771 (55.7)	21,307,587 (54.7)
Patient’s age (years)***								
<1	709,344 (2.0)	854,255 (2.0)	879,123 (2.0)	886,564 (1.9)	911,925 (1.9)	900,690 (1.8)	408,184 (1.1)	442,932 (1.2)
1 to 4.9	3,058,217 (8.5)	3,494,539 (8.3)	3,619,163 (8.1)	3,795,684 (8.0)	3,733,843 (7.7)	3,835,213 (7.6)	1,751,344 (4.7)	1,848,141 (4.8)
5 to 14.9	3,402,712 (9.5)	3,894,502 (9.3)	4,049,984 (9.1)	4,386,178 (9.2)	4,293,157 (8.8)	4,379,506 (8.7)	2,205,215 (5.9)	2,142,629 (5.5)
15 to 29.9	7,796,556 (21.8)	9,002,458 (21.4)	9,385,892 (21.0)	9,753,613 (20.5)	9,775,338 (20.1)	9,808,803 (19.5)	7,614,007 (20.4)	7,448,333 (19.3)
30 to 44.9	8,922,339 (24.9)	10,388,808 (24.7)	11,062,088 (24.7)	11,787,197 (24.7)	12,183,376 (25.1)	12,685,148 (25.3)	10,362,078 (27.8)	10,884,012 (28.2)
45 to 59.9	6,231,790 (17.4)	7,418,734 (17.6)	7,981,458 (17.8)	8,519,424 (17.9)	8,759,086 (18.0)	9,126,173 (18.2)	7,646,706 (20.5)	8,182,464 (21.2)
60+	5,732,859 (16.0)	7,029,558 (16.7)	7,752,723 (17.3)	8,532,576 (17.9)	8,941,365 (18.4)	9,460,669 (18.9)	7,359,823 (19.7)	7,698,928 (19.9)
Prescriber’s council								
CRM	33,702,721 (91 5)	39,339,662 (90.9)	41,724,263 (90.6)	44,310,145 (90.2)	45,023,321 (90.0)	46,671,793 (90.2)	34,032,944 (87.9)	34,953,230 (87.6)
CRMV	497,879 (1 4)	602,224 (1.4)	650,362 (1.4)	701,737 (1.4)	727,526 (1.5)	766,791 (1.5)	770,106 (2.0)	780,056 (2.0)
CRO	2,495,885 (6 8)	3,051,959 (7.1)	3,336,078 (7.2)	3,754,846 (7.7)	3,938,117 (7.9)	4,150,998 (8.0)	3,742,077 (9.7)	3,987,628 (10.0)
RMS	146,472 (0 4)	286,608 (0.7)	359,441 (0.8)	339,350 (0.7)	363,998 (0.7)	129,990 (0.3)	153,137 (0.4)	192,341 (0.5)

^!^The variable “prescriber’s council” did not show missing values. *Sales data for the months of September 2014 and July 202 are not available. **Missing values for the patient’s sex ranged from 1.65% in 2015 to 2.41% in 2020. ***Missing values for patient’s age ranged from 2.68% in 2014 to 3.49% in 2020.

Prescribers from the medical category accredited by the Regional Council of Medicine (CRM) were responsible for the majority of sales, representing between 87.6 and 91.5% in 2014 and 2021, respectively. Dentists, registered with the Regional Council of Dentistry (CRO), appear in smaller proportions, between 6.8 and 10% in 2014 and 2021, followed, in decreasing order, by veterinarians registered with the Regional Council of Veterinary Medicine (CRMV) and doctors registered with the Ministry of Health (RMS) (Table 2).

**Figure 2.**
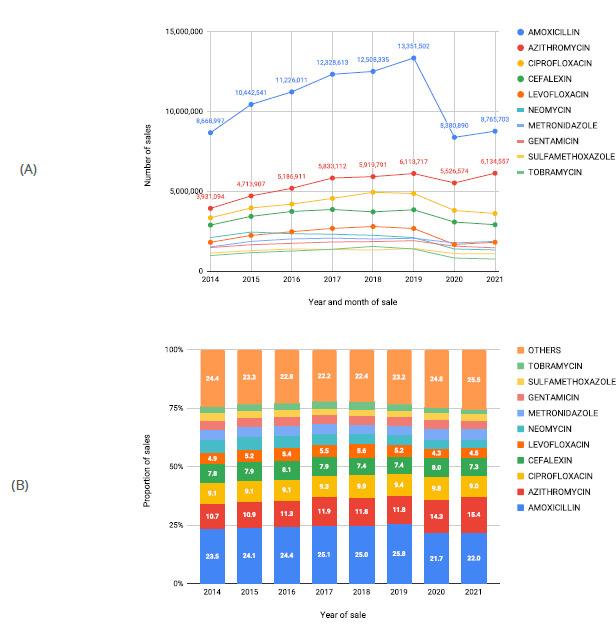
Sales of manufactured medicines containing an antimicrobial as an active ingredient, as recorded in the National System for the Management of Controlled Products (SNGPC), regarding the (A) number and (B) proportion represented by the ten main active ingredients sold, by year of sale, Brazil, 2014 to 2021*.

Note: Missing data for the months of September/2014 and July/2020.

The top ten active ingredients registered with SNGPC were in decreasing order: amoxicillin (85,672,592 sales), azithromycin (43,359,663), ciprofloxacin (33,272,063), cephalexin (27,463,286), levofloxacin (18,149,559), neomycin (16,279,510), metronidazole (15,176,847), gentamicin (13,492,715), sulfamethoxazole (10,103,194) and tobramycin (9,287,485). Together, these active ingredients account for 76.5% of total sales from 2014 to 2021. Amoxicillin was the best-selling active ingredient (24.1% of the total), with a growth trend from 2014 to 2019 (from 23.5 to 25.8%), followed by a reduction in 2020 (21.7%). Azithromycin, the second best-selling active ingredient, showed a modest increase between 2014 and 2019 (from 10.7 to 11.8%), with a sharper increase from 2020 (14.3%) to 2021 (15.4%), compared to previous years. An increase of more than 600 thousand sales of medicines containing azithromycin was observed in 2021 compared to 2020 (Figure 2A and 2B).

## DISCUSSION

The data analyzed in this study represent the scenario of antimicrobial sales recorded in SNGPC, during the period from 2014 to 2021 in Brazil, and their variations over time and between the different regions of the country. The importance of a comprehensive analysis that incorporates both observed and imputed data is emphasized, allowing a better understanding of sales trends and their implications for public health. This integrated approach is essential for the development of applicable policies that respond to changes in market behavior and emerging health needs.

A growing pattern in antimicrobial sales was observed in the period from 2014 to 2019, followed by a decrease in 2020, a trend similar to that observed in national and international literature, especially in low- and middle-income countries^8^ ^,^ ^18^ ^,^ ^19^ ^,^ ^20^. The most widely dispensed antibiotics in Brazil were amoxicillin, azithromycin, ciprofloxacin, and cephalexin. This consumption profile can be attributed to several factors, including the widespread prescription of these drugs for respiratory infections and common bacterial diseases such as pharyngitis, ear infections and urinary tract infections. Amoxicillin, for example, is frequently used for its safety and efficacy profile against a variety of pathogens, making it a popular choice among health care professionals^6^ ^,^ ^9^.

It is worth noting that 2020 was markedly affected by the COVID-19 pandemic. Previous studies have shown that the pandemic led to a reduction in sales of some categories of medicines, whether due to difficulties in accessing health services, interruption of production of the active ingredient, difficulties in transporting inputs due to restrictions imposed because of the pandemic and underreporting of sales in the system, or other factors^17^ ^,^ ^19^ ^,^ ^21^ ^,^ ^22^.

On the other hand, during the COVID-19 pandemic, there was a significant increase in the dispensing of azithromycin in Brazil, driven by the use of the “COVID-kit”, which included drugs such as azithromycin, chloroquine and ivermectin. The indiscriminate use of these drugs compromised the rational use of antimicrobials, favoring the development of AMR and the emergence of resistant strains. In addition, the lack of robust scientific evidence on their efficacy against the SARS-CoV-2 hampered the response to the pandemic and put the control of future infections at risk^18^ ^,^ ^23^. Therefore, continuous analysis of antimicrobial sales is crucial, since AMR is a growing concern worldwide, as highlighted by the World Health Organization^24^.

The drop in sales of all drugs in 2021 can be explained by the change in the mandatory registration with SNGPC, starting in October of that year, as pointed out in other similar studies on the registration of sales of antidepressants and antibiotics^19^ ^,^ ^25^ ^,^ ^26^. The deregulation of this registry is quite detrimental to pharmacovigilance in Brazil. Although Anvisa’s RDC No. 586/2021^25^ establishes that pharmacies must keep records of the movement in their internal records, for the purposes of proving stock and inspection, during the temporary suspension of the mandatory registration in SNGPC, this data ceased to form a national database, compromising the monitoring of sales of critical drugs^17^ ^,^ ^19^ ^,^ ^26^. In January 2025, Anvisa announced that SNGPC was fully operational and that establishments should prepare for the return of mandatory registration at the beginning of the year^27^. However, as of March 2025, no regulation had been published that resumed the mandatory registration of controlled drugs in SNGPC.

There was heterogeneity in the coverage of municipalities with sales registered in SNGPC, which suggests the existence of regional inequalities in access to antimicrobials, as well as in the supply of the system. Although some areas have shown progress, others still face substantial challenges that require specific interventions. The literature indicates that these inequalities can compromise the effectiveness of public health policies, requiring targeted strategies to ensure equitable and responsible access to medicines, in addition to adherence to SNGPC^11^ ^,^ ^23^.

The predominance of sales to women, higher than the proportion of women in the population, suggests that the prescription pattern may be influenced by demographic characteristics and by differences in the behavior of men and women in seeking health care. Furthermore, it is pertinent to note the significant participation of CRM in prescriptions, as well as of physicians without CRM but who were registered with the Ministry of Health. This registry was created by Law No. 12,871/2013 and Ordinance No. 2,477/2013, for application within the scope of the Mais Médicos Program, to authorize the work of professionals who did not yet have the necessary requirements to register with the CRM. These findings reinforce the importance of implementing educational interventions aimed at health professionals to improve the responsible use of antimicrobials, especially in vulnerable populations^8^ ^,^ ^11^ ^,^ ^28^.

Regarding age group, the higher consumption of antibiotics among individuals aged “60 or over” during the COVID-19 pandemic may be related to the change in the age profile of the Brazilian population, which is aging, and the higher prevalence of comorbidities at this stage of life. These conditions were associated with a higher risk and predisposition to developing severe cases of COVID-19, which led to a greater use of medications, including antibiotics^29^. On the other hand, the sharp reduction in antibiotic sales among children under 15 years of age may be related to the fact that children were less exposed to infections during the period of confinement and suspension of school activities, which limited the contagion and spread of diseases and, consequently, may have reduced the need for medical treatments for common respiratory infections in this age group^30^.

These facts highlight the need to implement public policies, educational and intervention strategies that promote the rational use of these drugs, aiming to improve public health outcomes and the economic impact on society and the Brazilian Unified Health System (SUS)^11^ ^,^ ^28^. In this context, the Stewardship Brazil Project, carried out in 2019, stands out, which evaluated the Antimicrobial Use Management Programs (PGUA) in the adult intensive care unit (ICU) in Brazilian hospitals, highlighting the importance of the rational and controlled use of antimicrobials to combat bacterial infections. It is therefore understood that the implementation of stewardship protocols, focusing on reducing the indiscriminate use of antibiotics and on accurate microbiological diagnosis, prevents AMR by optimizing treatment in the ICU^31^ ^,^ ^32^.

In view of this, we recognize the importance of similar strategies aimed at the community, considering that the rational use of antimicrobials outside the hospital environment is also a relevant challenge. Some international initiatives propose stewardship approaches aimed at primary care and self-medication, aiming to reduce the inappropriate use of these drugs in the general population, outside the hospital environment^33^ ^,^ ^34^.

The reduction in the number of antimicrobial sales records in SNGPC as of November 2021, as reported by the PBDA, is a matter of concern. The suspension of the mandatory data transmission by RDC 586/2021 is already producing significant gaps in the information available for analysis, compromising the ability to monitor trends and prescribing practices. This problem has been highlighted by other studies^17^ ^,^ ^18^ ^,^ ^19^ ^,^ ^22^ ^,^ ^26^, which highlighted the importance of a robust data collection system to monitor and understand actual drug consumption.

It is important to consider the limitations of this study, including the distinction between sales and actual drug consumption, which may not accurately reflect the reality of use. The literature suggests that sales records do not necessarily translate into actual consumption, which is a critical factor to be considered in future analyses^17^ ^,^ ^18^. However, during the period analyzed, the registration conditions were kept constant, so that the data for each year are comparable, which allows for the assessment of fluctuations and trends.

In summary, this study highlights the importance of continuous monitoring of antimicrobial sales in Brazil to understand consumption patterns, factors that influence prescription, and availability of these drugs. Detailed analysis of these data, including regional and demographic variations, can support regulatory policies and educational interventions aimed at the responsible use of these drugs. The suspension of the mandatory data transmission by SNGPC, as per RDC 586/2021, compromises the integrity of monitoring, which may negatively impact public health policies. Thus, it is urgent to reestablish a robust data registration system and implement educational strategies to combat AMR and improve health outcomes in the country.
