# Supplementary material for: Antimicrobial sales profile in Brazil from 2014 to 2021: analysis of records from the National System of Controlled Products Management
Source: Rev Bras Epidemiol. 2025 Aug 4;28:e250040. doi: 10.1590/1980-549720250040 (PMC12321154; doi:10.1590/1980-549720250040)
Supplement: Translation

# Translation: Perfil de venda de antimicrobianos no Brasil de 2014 a 2021: análise dos registros do Sistema Nacional de Gerenciamento de Produtos Controlados

**DOI:** 10.1590/1980-549720250040.2

**Authors:** Jakeline Ribeiro Barbosa, Giovanny Vinícius Araújo de França, Aurélio Matos Andrade, Beatriz Torres Araújo, Cláudio Maierovitch Pessanha Henriques, Mariana Pastorello Verotti

**Affiliations:** I Fundação Oswaldo Cruz, Universidade Aberta do Sistema Único de Saúde, Secretaria Executiva - Brasília (DF), Brasil.; II Ministério da Saúde, Secretaria de Ciência, Tecnologia e Inovação e do Complexo Econômico-Industrial da Saúde, Departamento de Ciência e Tecnologia - Brasília (DF), Brasil.; III Fundação Oswaldo Cruz, Programa de Evidências para Políticas e Tecnologias de Saúde - Brasília (DF), Brasil.; IV Fundação Oswaldo Cruz, Núcleo de Epidemiologia e Vigilância em Saúde - Brasília (DF), Brasil.

**Keywords:** Anti-infecciosos, Comercialização de produtos, Sistemas de informação, Brasil, Resistência microbiana a medicamentos

## Abstract

**Objetivo:**

Analisar o perfil de dispensação de antimicrobianos no Brasil durante o período de 2014 a 2021 com base nos registros do Sistema Nacional de Gerenciamento de Produtos Controlados (SNGPC) da Agência Nacional de Vigilância Sanitária (Anvisa).

**Métodos:**

Foi realizado um estudo ecológico descritivo, de série temporal, utilizando dados de farmácias e drogarias privadas, sobre a venda de medicamentos antimicrobianos. Foram incluídos medicamentos vendidos de janeiro de 2014 a novembro de 2021, com análise das variáveis: mês, ano, município, estado, princípio ativo, conselho de classe do prescritor, sexo e idade do paciente.

**Resultados:**

No período do estudo, foram registradas 532.518.866 vendas de medicamentos de controle especial no SNGPC, sendo 66,8% de antimicrobianos. Observou-se um aumento nas vendas até 2019, com queda em 2020, período da pandemia de COVID-19, e novo aumento em 2021. As regiões Sudeste e Nordeste concentraram as maiores vendas, com a Sudeste liderando. As vendas foram mais altas entre mulheres, especialmente na faixa etária de 30 a 44,9 anos, com um aumento de vendas em pacientes com 60 anos ou mais. Os antimicrobianos mais vendidos foram amoxicilina, azitromicina, ciprofloxacino e cefalexina.

**Conclusão:**

O estudo revela diferenças de consumo por região, gênero e idade, e destaca a importância de intervenções educativas para o uso responsável de antimicrobianos. Aponta ainda que a suspensão do registro obrigatório de dados no SNGPC pode comprometer o monitoramento necessário para combater a resistência bacteriana e melhorar a saúde pública no Brasil.

## INTRODUÇÃO

O uso de antimicrobianos é um marco na história da medicina, proporcionando tratamento eficaz para uma vasta gama de infecções, em especial as bacterianas, que, anteriormente, eram as causas mais comuns de morbidade e mortalidade^1^ ^,^ ^2^ ^,^ ^3^. No entanto, o crescimento alarmante da resistência a antimicrobianos (RAM) representa hoje uma das maiores ameaças à saúde global. A RAM não apenas limita as opções terapêuticas disponíveis, mas também eleva os custos da atenção à saúde e aumenta a carga de doenças em nível mundial^4^ ^,^ ^5^.

A situação do consumo de antimicrobianos varia significativamente ao redor do mundo, refletindo diferenças em práticas de prescrição, acesso a medicamentos e políticas de saúde^6^ ^,^ ^7^. Em países em desenvolvimento, como o Brasil, a questão é particularmente complexa devido à desigualdade socioeconômica e estrutural, além de desafios específicos relacionados à implementação de políticas de uso racional de antimicrobianos^8^ ^,^ ^9^.

No contexto brasileiro, a RAM tem se tornado uma preocupação crescente^10^ ^,^ ^11^ ^,^ ^12^. Estudos epidemiológicos têm demonstrado níveis preocupantes de bactérias resistentes a antimicrobianos comumente utilizados, como penicilinas e cefalosporinas, tanto em ambientes hospitalares quanto na comunidade, comprometendo a eficácia dos tratamentos, aumentando a gravidade das infecções e a duração da doença, assim como os custos de atenção à saúde^9^ ^,^ ^12^ ^,^ ^13^. A disseminação da RAM em estabelecimentos assistenciais de saúde, como hospitais e clínicas, assim como em comunidades, evidencia a necessidade urgente de estratégias coordenadas para controlar e mitigar esse problema de saúde pública^11^ ^,^ ^14^.

Nesse sentido, a partir de 2011, a Agência Nacional de Vigilância Sanitária (Anvisa) estabeleceu o controle de medicamentos à base de antimicrobianos, e em 2014 seu registro passou a ser obrigatório no Sistema Nacional de Gerenciamento de Produtos Controlados (SNGPC), assim como já era para os medicamentos sujeitos a controle especial. O SNGPC foi estabelecido para monitorar e regular a dispensação de medicamentos, incluindo antimicrobianos, em farmácias e drogarias privadas em todo o território nacional^15^ ^,^ ^16^.

Ao longo dos anos, o SNGPC tem desempenhado um papel crucial na regulamentação e monitoramento da dispensação de antimicrobianos no Brasil. Desde sua implementação, o SNGPC tem proporcionado uma infraestrutura potente para coleta e análise de dados sobre a distribuição e o uso desses medicamentos em farmácias e drogarias em todo o país. Esse sistema possibilita uma avaliação detalhada dos padrões de prescrição e dispensação, assim como a produção de informações essenciais para o desenvolvimento de políticas de saúde baseadas em evidências^17^ ^,^ ^18^ ^,^ ^19^.

A análise e monitoramento dos hábitos de prescrição e consumo de medicamentos antimicrobianos no país tem melhorado a compreensão das tendências de uso inadequado, tendo em vista que a Política Nacional de Medicamentos (PNM) ainda se encontra incipiente no âmbito da dispensação de antimicrobianos no Brasil. Isso interfere também nas políticas de saúde pública direcionadas à promoção do uso racional desses medicamentos, que contribuem com decisões regulatórias e ações educativas de uso prescricional. O presente estudo tem por objetivo analisar o perfil de dispensação de antimicrobianos no Brasil durante o período de 2014 a 2021.

## MÉTODOS

Foi realizado um estudo ecológico descritivo, de serie temporal, fundamentado nas bases de dados do SNGPC, disponibilizadas publicamente pela Anvisa por meio do Portal Brasileiro de Dados Abertos (PBDA), no endereço eletrônico: https://dados.gov.br/dados/organizacoes/visualizar/agencia-nacional-de-vigilancia-sanitaria-anvisa. Os dados deste estudo foram obtidos dos registros de dispensação de medicamentos industrializados do SNGPC, referente à classe dos antimicrobianos, provenientes de farmácias e drogarias privadas. A atualização mais recente do PBDA ocorreu em 8 de agosto de 2023, e os dados estão disponíveis sob licença *Creative Commons Attribution*.

Em consulta realizada no PBDA em 2 de março de 2024, foram identificados 96 arquivos, sendo:

- Arquivo com a documentação do sistema e dicionário de variáveis;
- Outros 95 arquivos em formato *Comma-Separated Values* (CSV), contendo vendas de medicamentos industrializados, mês a mês, no período de janeiro de 2014 a novembro de 2021.

De acordo com a Resolução de Diretoria Colegiada (RDC) 586/2021, os prazos para a transmissão dos dados de venda de medicamentos sujeitos ao controle especial para a base nacional do SNGPC foram temporariamente suspensos, passando a ser opcional a partir de 5 de outubro de 2021. A partir de 23 de dezembro de 2022, o acesso ao sistema foi interrompido totalmente, com a justificativa de que seria implementada uma solução tecnológica. Dessa forma, no período considerado para análise, de 1 de janeiro de 2024 a 30 de novembro de 2021, os dados dos meses de setembro de 2014 e julho de 2020 não se encontram disponíveis.

Os 95 arquivos foram extraídos individualmente do site do PBDA, no formato “.csv” e convertidos para o formato “*.dta”, de modo a possibilitar a análise utilizando-se o *software Stata* ^®^ versão 12.0. Em cada arquivo, foram selecionados os registros com a variável tipo de receituário igual a “5 - Receita Antimicrobiano em 2 vias”, de modo a manter apenas as vendas de antimicrobianos. Posteriormente, os arquivos foram agrupados por ano, a fim de otimizar o processamento e a análise.

Foram consideradas as seguintes variáveis: mês e ano da venda do medicamento; município e Unidade Federativa do endereço da farmácia ou drogaria onde ocorreu a venda; nome do princípio ativo do medicamento industrializado, conforme cadastro na Anvisa; conselho de classe do profissional que prescreveu o medicamento vendido; Unidade Federativa do conselho de classe do profissional prescritor; sexo do paciente (masculino ou feminino); e idade do paciente em meses ou anos.

Em relação aos dados faltantes dos meses de setembro de 2014 e julho de 2020, foi utilizado o comando *ipolate* do *software Stata* ^®^, que considera a série histórica e realiza a imputação utilizando a interpolação linear para estimar os pontos faltantes, com base nos pontos conhecidos da série. Considerando que o envio de dados para o SNGPC passou a ser opcional a partir de 5 de outubro de 2021, utilizou-se a mesma técnica para imputar valores para o mês de novembro de 2021.

Este estudo foi realizado de acordo com as Resoluções nº. 466/2012 e 580/2018 do Conselho Nacional de Saúde (CNS). Essa pesquisa faz parte de um projeto maior, intitulado “Cenário Brasileiro de Uso de Antimicrobianos e o Perfil de Resistência Identificado no SUS”, que obteve aprovação do Comitê de Ética em Pesquisa sob CAAE 42456920.6.0000.8027 e financiamento pelo Conselho Nacional de Desenvolvimento Científico e Tecnológico (CNPq)/Gerência Regional de Brasília/Fiocruz (edital 41/2018, Processo nº 440206/2019-7).

### Declaração de disponibilidade de dados

Os dados estão disponíveis publicamente no Portal de Dados Abertos, pelo site: https://dados.gov.br/dados/conjuntos-dados/venda-de-medicamentos-controlados-e-antimicrobianos---medicamentos-industrializados.

## RESULTADOS

No período de janeiro de 2014 a novembro de 2021 foram registradas 532.518.866 vendas de medicamentos industrializados de controle especial e antimicrobianos no SNGPC. Desses, 355.683.685 tinham tipo de receituário igual a “5 - Receita Antimicrobiano em 2 vias”, correspondendo a 66,8% do total. A Figura 1 apresenta a série histórica de vendas de antimicrobianos por ano e mês da venda, total (A) e estratificada por região (B), bem como a série com o consolidado de vendas por ano e região (C). Nos gráficos, é possível comparar os dados brutos obtidos em relação às séries considerando as imputações realizadas.

**Figura 1.** Número de vendas de medicamentos industrializados contendo um antimicrobiano como princípio ativo, (A) por ano/mês da venda, (B) por ano/mês da venda e região e (C) por ano da venda e região, comparando-se a série com valores observados com a série incluindo valores imputados, Brasil, 2014 a 2021*.

*Não estão disponíveis dados de vendas referentes aos meses de setembro de 2014 e julho de 2020.

Observa-se tendência linear de crescimento nas vendas ao longo dos anos de 2014 a 2019 e aparente sazonalidade, com aumento notável no meio do ano nas regiões Sul e Sudeste, durante o inverno, e redução em todas as regiões no início de cada ano. O mencionado crescimento até 2019 foi interrompido, com uma queda acentuada em 2020, ano em que começou a pandemia de COVID-19. Em novembro de 2021, os dados imputados sugerem que os dados observados nesse período foram bastante subestimados, sendo esperada uma ligeira queda nos meses de novembro e dezembro, seguindo a tendência de diminuição das vendas observada a partir de junho de 2021 (Figura 1A).

Destacam-se as regiões Sudeste e Nordeste, que concentraram a maior parte das vendas no período analisado, apontando a região Sudeste, em primeiro lugar, sendo consistentemente superior às demais regiões em toda a série (Figuras 1B e 1C). A ordenação dos volumes de vendas por região geográfica corresponde diretamente à ordem de número de habitantes. Considerando-se os dados agregados por ano e por região, notam-se padrões diferentes nas séries com e sem valores imputados, especialmente nas regiões Sudeste e Nordeste. Com base nas análises de imputação, observa-se uma queda brusca das vendas em 2020 e uma tendência de incremento importante das vendas no ano de 2021, o que não foi possível identificar por meio dos dados brutos.

As regiões Sudeste e Sul apresentaram elevadas proporções de municípios com registro de vendas no SNGPC em todo o período, com pequena variabilidade. Os estados do Espírito Santo e do Rio de Janeiro alcançaram 100% dos municípios com registro em 2021; o Rio de Janeiro apresentou cobertura de 100% entre os anos de 2015 e 2021. A região Centro-Oeste também apresentou elevada cobertura, porém com maior variabilidade, passando de 87,0% em 2014 para 95,0% em 2021. Os estados de Goiás, Mato Grosso do Sul e Mato Grosso apresentaram coberturas homogêneas entre si ao longo da série (Tabela 1).

**Tabela 1.** Número total e percentual de municípios de cada Unidade da Federação (UF) que registraram vendas de antimicrobianos no Sistema Nacional de Gerenciamento de Produtos Controlados (SNGPC), estratificados por ano, Brasil, 2014 a 2021.

Região/UF	Total de Municípios	2014		2015		2016		2017		2018		2019		2020		2021	
		n	%	n	%	n	%	n	%	n	%	n	%	n	%	n	%
Norte	450	200	44,4	198	44,0	215	47,8	225	50,0	232	51,6	251	55,8	252	56,0	275	61,1
Acre	22	18	81,8	18	81,8	17	77,3	18	81,8	17	77,3	17	77,3	17	77,3	18	81,8
Amapá	16	2	12,5	2	12,5	2	12,5	8	50,0	5	31,3	5	31,3	3	18,8	4	25,0
Amazonas	62	11	17,7	10	16,1	12	19,4	11	17,7	10	16,1	15	24,2	16	25,8	20	32,3
Pará	144	68	47,2	64	44,4	65	45,1	64	44,4	66	45,8	70	48,6	68	47,2	74	51,4
Rondônia	52	46	88,5	45	86,5	47	90,4	48	92,3	49	94,2	50	96,2	51	98,1	51	98,1
Roraima	15	5	33,3	6	40,0	8	53,3	9	60,0	10	66,7	10	66,7	11	73,3	13	86,7
Tocantins	139	50	36,0	53	38,1	64	46,0	67	48,2	75	54,0	84	60,4	86	61,9	95	68,3
Nordeste	1.794	1.233	68,7	1.282	71,5	1.309	73,0	1.346	75,0	1.348	75,1	1.371	76,4	1.411	78,7	1.437	80,1
Alagoas	102	65	63,7	73	71,6	77	75,5	81	79,4	83	81,4	87	85,3	87	85,3	88	86,3
Bahia	417	301	72,2	324	77,7	342	82,0	348	83,5	359	86,1	370	88,7	375	89,9	375	89,9
Ceará	184	145	78,8	144	78,3	146	79,3	149	81,0	149	81,0	146	79,3	153	83,2	156	84,8
Maranhão	217	107	49,3	106	48,8	103	47,5	112	51,6	111	51,2	111	51,2	114	52,5	118	54,4
Paraíba	223	175	78,5	187	83,9	187	83,9	196	87,9	196	87,9	197	88,3	202	90,6	210	94,2
Pernambuco	185	158	85,4	155	83,8	155	83,8	159	85,9	159	85,9	162	87,6	162	87,6	161	87,0
Piauí	224	100	44,6	102	45,5	99	44,2	97	43,3	87	38,8	91	40,6	107	47,8	111	49,6
Rio Grande do Norte	167	131	78,4	138	82,6	145	86,8	148	88,6	149	89,2	149	89,2	148	88,6	152	91,0
Sergipe	75	51	68,0	53	70,7	55	73,3	56	74,7	55	73,3	58	77,3	63	84,0	66	88,0
Sudeste	1.668	1.543	92,5	1.571	94,2	1.588	95,2	1.600	95,9	1.601	96,0	1.611	96,6	1.615	96,8	1.621	97,2
Espírito Santo	78	74	94,9	74	94,9	74	94,9	75	96,2	76	97,4	77	98,7	78	100,0	78	100,0
Minas Gerais	853	776	91,0	792	92,8	804	94,3	813	95,3	819	96,0	825	96,7	825	96,7	829	97,2
Rio de Janeiro	92	91	98,9	92	100,0	92	100,0	92	100,0	92	100,0	92	100,0	92	100,0	92	100,0
São Paulo	645	602	93,3	613	95,0	618	95,8	620	96,1	614	95,2	617	95,7	620	96,1	622	96,4
Centro-Oeste	501	436	87,0	447	89,2	451	90,0	454	90,6	462	92,2	470	93,8	471	94,0	476	95,0
Distrito Federal*	35	21	60,0	22	62,9	22	62,9	22	62,9	22	62,9	23	65,7	26	74,3	26	74,3
Goiás	246	218	88,6	224	91,1	230	93,5	230	93,5	233	94,7	238	96,7	237	96,3	239	97,2
Mato Grosso	141	127	90,1	132	93,6	129	91,5	131	92,9	134	95,0	135	95,7	134	95,0	138	97,9
Mato Grosso do Sul	79	70	88,6	69	87,3	70	88,6	71	89,9	73	92,4	74	93,7	74	93,7	73	92,4
Sul	1.191	1.099	92,3	1.117	93,8	1.124	94,4	1.140	95,7	1.140	95,7	1.146	96,2	1.146	96,2	1.155	97,0
Paraná	399	373	93,5	375	94,0	378	94,7	386	96,7	384	96,2	382	95,7	382	95,7	388	97,2
Rio Grande do Sul	497	454	91,3	466	93,8	466	93,8	468	94,2	470	94,6	472	95,0	474	95,4	475	95,6
Santa Catarina	295	272	92,2	276	93,6	280	94,9	286	96,9	286	96,9	292	99,0	290	98,3	292	99,0

*O Distrito Federal (DF) é dividido em regiões administrativas e não em municípios.

A região Norte foi a que apresentou menores coberturas, variando de 44,4% em 2014 a 61,1% em 2021. Os estados com menos registros de vendas de antimicrobianos no SNGPC foram Amapá e o Amazonas, onde 25 e 32,3% dos municípios obtiveram registros de venda em 2021, respectivamente. O estado de Roraima apresentou um incremento importante no número de municípios com registros, passando de 5 em 2014 para 13 em 2021 do total de 15 municípios (Tabela 1).

As vendas foram maiores para pessoas do sexo feminino, no período de 2014-2021, com percentuais variando de 55,1% a 55,7%. A distribuição das vendas por idade do paciente foi predominante na faixa etária de 30 a 44,9 anos, variando entre 24,9% em 2014 e 28,2% em 2021. A categoria “60 anos ou mais” apresentou um crescimento do consumo, ao longo dos anos, passando de 16,0% em 2014 para 19,9% em 2021 que corresponde, aproximadamente, à proporção de pessoas dessa faixa na população. A faixa etária de menores de 15 anos foi a que apresentou diminuição mais acentuada de prescrições entre 2019 e 2020, com discreto aumento em 2021 em relação ao ano anterior (Tabela 2).

**Tabela 2.** Número de vendas de medicamentos industrializados contendo um antimicrobiano como princípio ativo por ano da venda, segundo conselho do prescritor, sexo e idade do paciente, Brasil, 2014 a 2021^!^.

Variáveis	Ano da venda							
	2014*	2015	2016	2017	2018	2019	2020*	2021
	n (%)	n (%)	n (%)	n (%)	n (%)	n (%)	n (%)	n (%)
Sexo do paciente**								
Masculino	16.263.873 (44,9)	19.145.755 (45,0)	20.266.780 (44,8)	21.499.749 (44,6)	21.838.691 (44,4)	22.702.196 (44,7)	16.719.276 (44,3)	17.680.913 (45,4)
Feminino	19.967.350 (55,1)	23.419.952 (55,0)	25.021.209 (55,3)	26.744.166 (55,4)	27.313.786 (55,6)	28.060.636 (55,3)	21.044.771 (55,7)	21.307.587 (54,7)
Idade do paciente (anos)***								
<1	709.344 (2,0)	854.255 (2,0)	879.123 (2,0)	886.564 (1,9)	911.925 (1,9)	900.690 (1,8)	408.184 (1,1)	442.932 (1,2)
1 a 4,9	3.058.217 (8,5)	3.494.539 (8,3)	3.619.163 (8,1)	3.795.684 (8,0)	3.733.843 (7,7)	3.835.213 (7,6)	1.751.344 (4,7)	1.848.141 (4,8)
5 a 14,9	3.402.712 (9,5)	3.894.502 (9,3)	4.049.984 (9,1)	4.386.178 (9,2)	4.293.157 (8,8)	4.379.506 (8,7)	2.205.215 (5,9)	2.142.629 (5,5)
15 a 29,9	7.796.556 (21,8)	9.002.458 (21,4)	9.385.892 (21,0)	9.753.613 (20,5)	9.775.338 (20,1)	9.808.803 (19,5)	7.614.007 (20,4)	7.448.333 (19,3)
30 a 44,9	8.922.339 (24,9)	10.388.808 (24,7)	11.062.088 (24,7)	11.787.197 (24,7)	12.183.376 (25,1)	12.685.148 (25,3)	10.362.078 (27,8)	10.884.012 (28,2)
45 a 59,9	6.231.790 (17,4)	7.418.734 (17,6)	7.981.458 (17,8)	8.519.424 (17,9)	8.759.086 (18,0)	9.126.173 (18,2)	7.646.706 (20,5)	8.182.464 (21,2)
60+	5.732.859 (16,0)	7.029.558 (16,7)	7.752.723 (17,3)	8.532.576 (17,9)	8.941.365 (18,4)	9.460.669 (18,9)	7.359.823 (19,7)	7.698.928 (19,9)
Conselho do prescritor								
CRM	33.702.721 (91,5)	39.339.662 (90,9)	41.724.263 (90,6)	44.310.145 (90,2)	45.023.321 (90,0)	46.671.793 (90,2)	34.032.944 (87,9)	34.953.230 (87,6)
CRMV	497.879 (1,4)	602.224 (1,4)	650.362 (1,4)	701.737 (1,4)	727.526 (1,5)	766.791 (1,5)	770.106 (2,0)	780.056 (2,0)
CRO	2.495.885 (6,8)	3.051.959 (7,1)	3.336.078 (7,2)	3.754.846 (7,7)	3.938.117 (7,9)	4.150.998 (8,0)	3.742.077 (9,7)	3.987.628 (10,0)
RMS	146.472 (0,4)	286.608 (0,7)	359.441 (0,8)	339.350 (0,7)	363.998 (0,7)	129.990 (0,3)	153.137 (0,4)	192.341 (0,5)

^!^A variável “conselho do prescritor” não apresentou valores faltantes; *Não estão disponíveis dados de vendas referentes aos meses de setembro de 2014 e julho de 2020; **Valores faltantes para o sexo do paciente variaram entre 1,65% em 2015 e 2,41% em 2020; ***Valores faltantes para a idade do paciente variaram entre 2,68% em 2014 e 3,49% em 2020.

Prescritores da categoria médica credenciados junto ao Conselho Regional de Medicina (CRM) foram responsáveis pela maior parte das vendas, representando entre 87,6% e 91,5% em 2014 e 2021, respectivamente. Os dentistas, registrados no Conselho Regional de Odontologia (CRO), aparecem em proporções menores, entre 6,8 e 10% em 2014 e 2021, seguidos, em ordem decrescente, por médicos veterinários registrados no Conselho Regional de Medicina Veterinária (CRMV) e médicos com Registro do Ministério da Saúde (RMS) (Tabela 2).

Os dez principais princípios ativos registrados no SNGPC foram, em ordem decrescente: amoxicilina (85.672.592 de vendas), azitromicina (43.359.663), ciprofloxacino (33.272.063), cefalexina (27.463.286), levofloxacino (18.149.559), neomicina (16.279.510), metronidazol (15.176.847), gentamicina (13.492.715), sulfametoxazol (10.103.194) e tobramicina (9.287.485). Juntos, esses princípios ativos correspondem a 76,5% do total de vendas no período de 2014 a 2021. A amoxicilina foi o princípio ativo mais vendido (24,1% do total), com uma tendência de crescimento no período de 2014 a 2019 (de 23,5 para 25,8%), seguido de uma redução em 2020 (21,7%). A azitromicina, segundo princípio ativo mais vendido, apresentou um modesto incremento entre 2014 e 2019 (de 10,7 para 11,8%), com um aumento mais acentuado de 2020 (14,3%) e 2021, (15,4%), se comparado aos anos anteriores. Foi possível observar um incremento de mais de 600 mil vendas de medicamentos contendo azitromicina em 2021 comparação a 2020 (Figuras 2A e 2B).

**Figura 2.** Vendas de medicamentos industrializados contendo um antimicrobiano como princípio ativo, conforme registrado no Sistema Nacional de Gestão de Produtos Controlados (SNGPC), quanto ao (A) número e (B) proporção representados pelos dez principais princípios ativos vendidos, por ano da venda, Brasil, 2014 a 2021*.

Nota: Dados faltantes para os meses de setembro/2014 e julho/2020.

## DISCUSSÃO

Os dados analisados neste estudo representam o cenário das vendas de antimicrobianos registradas no SNGPC, durante o período de 2014 a 2021 no Brasil, e suas variações ao longo do tempo e entre as diferentes regiões do país. Ressalta-se a importância de uma análise abrangente, que incorpore tanto os dados observados quanto os imputados, permitindo uma melhor compreensão das tendências de vendas e suas implicações para a saúde pública. Essa abordagem integrada é fundamental para o desenvolvimento de políticas aplicáveis que respondam às mudanças no comportamento do mercado e às necessidades emergentes em saúde.

Constatou-se um padrão crescente nas vendas de antimicrobianos no período de 2014 a 2019, seguido por um decréscimo em 2020, tendência semelhante à observada na literatura nacional e internacional, especialmente em países de baixa e média renda^8^ ^,^ ^18^ ^,^ ^19^ ^,^ ^20^. Os antibióticos de maior dispensação no Brasil foram a amoxicilina, azitromicina, ciprofloxacino e cefalexina. Esse perfil de consumo pode ser atribuído a vários fatores, incluindo a ampla prescrição desses medicamentos em casos de infecções respiratórias e doenças bacterianas comuns, como faringites, otites e infecções urinárias. A amoxicilina, por exemplo, é frequentemente utilizada por seu perfil de segurança e eficácia contra uma variedade de patógenos, o que a torna uma escolha popular entre os profissionais de saúde^6^ ^,^ ^9^.

Vale destacar que o ano de 2020 foi marcadamente afetado pela pandemia de COVID-19. Estudos anteriores demonstraram que a pandemia levou a uma redução nas vendas de algumas categorias de medicamentos, seja por dificuldades no acesso aos serviços de saúde, interrupção da produção do princípio ativo, dificuldades no transporte de insumos pelas restrições impostas por causa da pandemia e subnotificação das vendas no sistema, ou outros fatores^17^ ^,^ ^19^ ^,^ ^21^ ^,^ ^22^.

Por outro lado, durante a pandemia de COVID-19, houve um aumento significativo na dispensação de azitromicina no Brasil, impulsionado pelo uso do “kit-COVID”, que incluía medicamentos como azitromicina, cloroquina e ivermectina. Esse cenário comprometeu o uso racional de antimicrobianos, favorecendo o desenvolvimento da RAM e o surgimento de cepas resistentes. Além disso, a falta de evidências científicas robustas sobre sua eficácia contra o SARS-CoV-2 prejudicou a resposta à pandemia e colocou em risco o controle de infecções futuras^18^ ^,^ ^23^. Portanto, a análise contínua das vendas de antimicrobianos é crucial, uma vez que a RAM é uma preocupação crescente em todo o mundo, conforme destacado pela Organização Mundial da Saúde^24^.

A queda nas vendas de todos os medicamentos em 2021 pode ser explicada pela mudança na obrigatoriedade de registro no SNGPC, a partir de outubro daquele ano, conforme apontado em outros estudo similares sobre o registro de vendas de antidepressivos e antibióticos^19^ ^,^ ^25^ ^,^ ^26^. A desregulamentação desse registro é bastante prejudicial para a farmacovigilância no Brasil. Apesar de a RDC nº 586/2021^25^ da Anvisa estabelecer que as farmácias devem manter a escrituração da movimentação nos registros internos, para fins de comprovação de estoque e fiscalização, durante a suspensão temporária da obrigatoriedade de registro no SNGPC, esses dados deixaram de compor uma base nacional, comprometendo o monitoramento das vendas de medicamentos críticos^17^ ^,^ ^19^ ^,^ ^26^. Em janeiro de 2025, a Anvisa noticiou que o SNGPC estava em plena operação e que os estabelecimentos deveriam se preparar para o retorno do registro obrigatório no início do ano^27^. No entanto, até março de 2025, não havia sido publicada nenhuma normativa que retomasse o registro compulsório de medicamentos controlados no SNGPC.

Verificou-se heterogeneidade na cobertura de municípios com vendas registradas no SNGPC, o que sugere a existência de desigualdades regionais no acesso a antimicrobianos, assim como na alimentação do sistema. Apesar de algumas áreas apresentarem avanços, outras ainda enfrentam desafios substanciais que demandam intervenções específicas. A literatura aponta que essas desigualdades podem comprometer a eficácia das políticas de saúde pública, exigindo estratégias direcionadas para garantir acesso equitativo e responsável aos medicamentos, além de adesão ao SNGPC^11^ ^,^ ^23^.

A predominância de vendas para mulheres, superior à proporção de mulheres na população, sugere que o padrão de prescrição pode estar influenciado por características demográficas e por diferenças entre o comportamento de homens e mulheres na busca por assistência à saúde. Além disso, é pertinente observar a significativa participação do CRM nas prescrições, bem como de médicos sem CRM, mas que tinham o registro do Ministério da Saúde. Esse registro foi criado por meio da Lei nº 12.871/2013 e da Portaria nº 2.477/2013, para aplicação no âmbito do Programa Mais Médicos, para autorizar o trabalho de profissionais que ainda não tinham os requisitos necessários para se inscrever no CRM. Esses achados reforçam a importância de implementar intervenções educacionais direcionadas a profissionais de saúde para melhorar o uso responsável de antimicrobianos, especialmente em populações vulneráveis^8^ ^,^ ^11^ ^,^ ^28^.

Em relação à faixa etária, o maior consumo de antibióticos entre indivíduos de “60 anos ou mais” durante a pandemia de Covid-19 pode estar relacionado à mudança no perfil etário da população brasileira, que está envelhecendo, e à maior prevalência de comorbidades nessa fase da vida. Essas condições foram associadas ao maior risco e à predisposição para o desenvolvimento de casos graves de Covid-19, o que levou a um maior uso de medicamentos, incluindo antibióticos^29^. Por outro lado, a redução acentuada das vendas de antibióticos entre menores de 15 anos pode estar relacionada ao fato de que as crianças ficaram menos expostas a infecções durante o período de confinamento e suspensão das atividades escolares, o que limitou o contágio e a propagação de doenças e, consequentemente, pode ter reduzido a necessidade de tratamentos médicos para infecções respiratórias comuns nessa faixa etária^30^.

Tais fatos evidenciam a necessidade de implementar políticas públicas, estratégias educacionais e de intervenção que promovam o uso racional desses medicamentos, visando melhorar os desfechos de saúde pública e o impacto econômico na sociedade e no Sistema Único de Saúde (SUS) do Brasil^11^ ^,^ ^28^. Nesse contexto, destaca-se o Projeto *Stewardship* Brasil, realizado em 2019, que avaliou os Programas de Gerenciamento do Uso de Antimicrobianos (PGUA) em unidades de terapia intensiva (UTI) de adultos dos hospitais brasileiros, destacando a importância do uso racional e controlado de antimicrobianos para combater infecções bacterianas. Compreende-se, portanto, que a implementação de protocolos de *stewardship*, com foco na redução do uso indiscriminado de antibióticos e no diagnóstico microbiológico preciso, previne a RAM otimizando o tratamento em UTI^31^ ^,^ ^32^.

Diante disso, reconhecemos a importância de estratégias semelhantes voltadas para a comunidade, considerando que o uso racional de antimicrobianos fora do ambiente hospitalar também é um desafio relevante. Algumas iniciativas internacionais propõem abordagens de *Stewardship* voltadas para a atenção primária e a automedicação, visando reduzir o uso inadequado desses medicamentos na população em geral, fora do ambiente hospitalar^33^ ^,^ ^34^.

A redução no número de registros de vendas dos antimicrobianos no SNGPC a partir de novembro de 2021, conforme informado pelo PBDA, é uma questão preocupante. A suspensão da obrigatoriedade de transmissão de dados pela RDC 586/2021 já está produzindo lacunas importantes nas informações disponíveis para análise, comprometendo a capacidade de monitorar tendências e práticas de prescrição. Esse problema foi apontado por outros estudos^17^ ^,^ ^18^ ^,^ ^19^ ^,^ ^22^ ^,^ ^26^, os quais ressaltaram a importância de um sistema robusto de coleta de dados para monitorar e entender o consumo real de medicamentos.

É importante considerar as limitações deste estudo, incluindo a distinção entre vendas e consumo real de medicamentos, que pode não refletir com precisão a realidade do uso. A literatura sugere que o registro de vendas não traduz necessariamente o consumo efetivo, o que é um fator crítico a ser considerado em análises futuras^17^ ^,^ ^18^. Entretanto, durante o período analisado, as condições de registro foram mantidas constantes, de forma que os dados de cada ano são comparáveis, o que permite avaliar oscilações e tendências.

Em suma, o presente estudo evidencia a importância do monitoramento contínuo das vendas de antimicrobianos no Brasil, a fim de compreender os padrões de consumo, os fatores que influenciam a prescrição e a disponibilidade desses medicamentos. A análise detalhada desses dados, incluindo variações regionais e demográficas, pode apoiar políticas regulatórias e intervenções educacionais voltadas ao uso responsável desses medicamentos. A suspensão da obrigatoriedade de transmissão de dados pelo SNGPC, conforme a RDC 586/2021, compromete a integridade do monitoramento, o que pode impactar negativamente as políticas de saúde pública. Assim, é urgente restabelecer um sistema robusto de registro de dados e implementar estratégias educativas para combater a RAM e melhorar os resultados em saúde no país.
